# Spreading of SARS-CoV-2 in West Africa and assessment of risk factors

**DOI:** 10.1017/S0950268820002149

**Published:** 2020-09-14

**Authors:** B. Tinto, S. Salinas, A. Dicko, T. S. Kagone, I. Traore, N. de Rekeneire, B. W. Bicaba, H. Hien, N. Meda, P. van de Perre, D. Kania, Y. Simonin

**Affiliations:** 1 Laboratoire National de Référence des Fièvres Hémorragiques Virales, Centre MURAZ, Institut National de Santé Publique (INSP), Bobo-Dioulasso, Burkina Faso; 2 Pathogenesis and Control of Chronic Infections, Université de Montpellier, INSERM, EFS, Montpellier, France; 3 Laboratoire Central de Référence, INSP, Ouagadougou, Burkina Faso; 4 Centre Muraz, INSP, Bobo-Dioulasso, Burkina Faso; 5 Expertise France, Paris, France; 6 Centre des Opérations de Réponse aux Urgences Sanitaires (CORUS), INSP, Ouagadougou, Burkina Faso; 7 INSP, Ouagadougou, Burkina Faso; 8 UFR/SDS, Université Ouaga I Professeur Joseph KI-ZERBO, Ouagadougou, Burkina Faso

**Keywords:** COVID-19, risk factor, SARS-CoV-2, spreading, West Africa

## Abstract

Although the African continent is, for the moment, less impacted than the rest of the world, it still faces the risk of a spread of COVID-19. In this study, we have conducted a systematic review of the information available in the literature in order to provide an overview of the epidemiological and clinical features of COVID-19 pandemic in West Africa and of the impact of risk factors such as comorbidities, climatic conditions and demography on the pandemic. Burkina Faso is used as a case study to better describe the situation in West Africa. The epidemiological situation of COVID-19 in West Africa is marked by a continuous increase in the numbers of confirmed cases. This geographic area had on 29 July 2020, 131 049 confirmed cases by polymerase chain reaction, 88 305 recoveries and 2102 deaths. Several factors may influence the SARS-CoV-2 circulation in Africa: (i) comorbidities: diabetes mellitus and high blood pressure could lead to an increase in the number of severe cases of SARS-CoV-2; (ii) climatic factors: the high temperatures could be a factor contributing to slow the spread of the virus and (iii) demography: the West Africa population is very young and this could be a factor limiting the occurrence of severe forms of SARS-CoV-2 infection. Although the spread of the SARS-CoV-2 epidemic in West Africa is relatively slow compared to European countries, vigilance must remain. Difficulties in access to diagnostic tests, lack of hospital equipment, but also the large number of people working in the informal sector (such as trading, businesses, transport and restoration) makes it difficult to apply preventive measures, namely physical distancing and containment.

## Introduction

On 31 December 2019, the city of Wuhan (Hubei Province, China), reported the emergence of an atypical pneumonia caused by a new coronavirus, later known as SARS-CoV-2 [1, 2]. Coronaviruses are a large family of RNA viruses that can cause a wide variety of diseases in animals and humans, from the common cold to SARS (Severe Acute Respiratory Syndrome), [3]. Coronaviruses are divided into four genera according to phylogeny: alpha-CoV, beta-CoV, gamma-CoV and delta-CoV [4]. Human coronaviruses (HCoVs) were first described in the 1960s in patients with cold [4]. Since then, six HCoVs have been discovered: 229E, OC43, NL63, HKU1, SARS-CoV-1 and MERS-CoV [4, 5]. Similar to SARS-CoV-1 and MERS-CoV, SARS-CoV-2 belongs to the genus beta-CoV (beta-coronavirus) [6]. On 7 January, Chinese researchers isolated the new SARS-CoV-2 from patients with pneumonia. From 13 January, the epidemic began to spread outside of China [7] and on 30 January 2020, the World Health Organization (WHO) Director-General declared the COVID-19 epidemic a public health emergency of international concern [8]. As of 29 July 2020, WHO has reported 16 558 289 confirmed cases and 656 093 deaths worldwide [9]. On 14 February 2020, the first case of COVID-19 on the African continent was registered in Egypt [10]. Since then, the disease has spread to several other African countries. In sub-Saharan Africa, Nigeria was the first country to report a case of COVID-19 on 27 February 2020 [11]. Nowadays, all the countries of the sub-Saharan zone have been affected by the pandemic. As of 29 July 2020, Nigeria is the most affected country by the coronavirus pandemic in West Africa followed by Ghana [12]. Previous studies have shown the circulation of MERS-CoV in camels in some West African countries (Nigeria, Burkina Faso and Mali) [13, 14]. However, no case of HCoVs has been reported until the appearance of SARS-CoV-2. Although the African continent is, for the moment, less impacted than the rest of the world, it still faces the risk of a spread of COVID-19. Even though most African countries repeatedly face epidemics and health crises which allowed them to learn from past outbreaks, many gaps remain in the patient care systems of many countries. The WHO therefore ‘fears Africa may not be able to face this pandemic, and calls on states to make their arrangements timely’ [15]. The transmission and spread of viruses can be influenced by a number of factors, including climatic conditions (such as temperature and humidity), population density and the quality of medical care [16, 17]. In this study we have conducted a systematic review of the information available in the literature in order to provide an overview of the epidemiological and clinical features of COVID-19 pandemic in West Africa and the impact of risk factors such as comorbidities, climatic conditions and demography on the pandemic. We focused our attention on Burkina Faso as it is one of the countries of West Africa with the most data available. Otherwise the way of life, the demography and the climate are comparable to most countries in West Africa.

## Methods

**Fig. 1.**
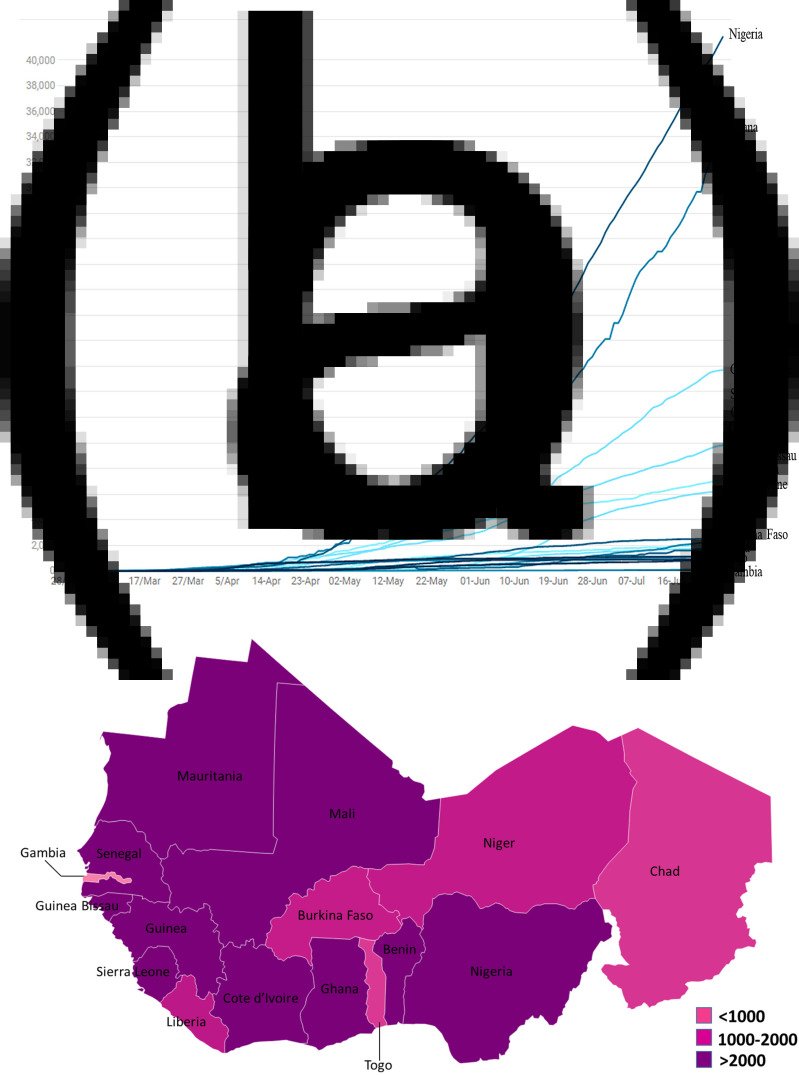
(a) Evolution of confirmed cases per day of the SARS-CoV-2 disease in West Africa from 28 February to 29 July 2020. (b) Mapping of the West Africa countries affected by SARS-CoV-2 as of 29 July 2020.

**Fig. 2.**
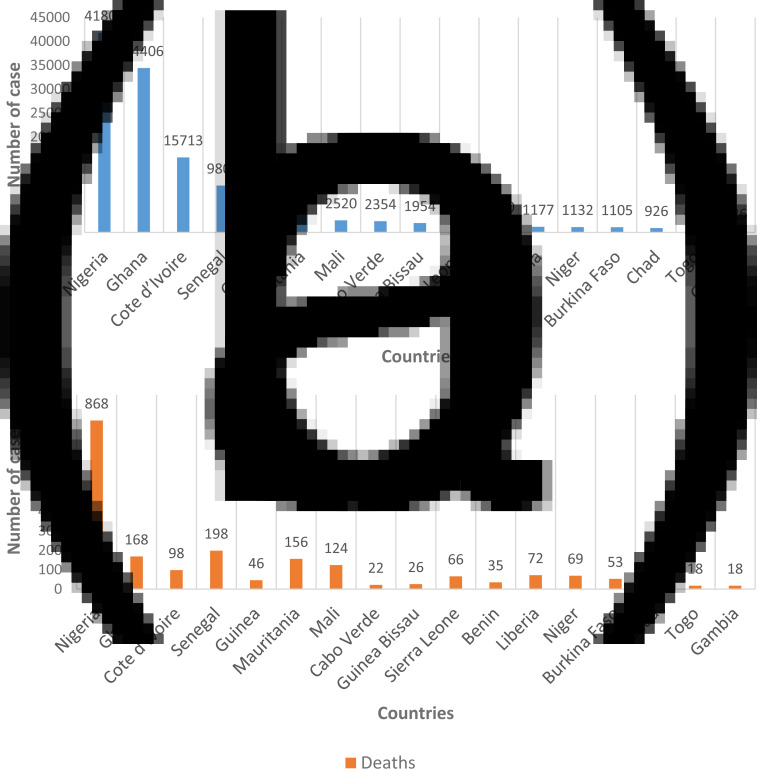
(a) Geographical distribution of confirmed cases of the SARS-CoV-2 disease in West Africa as 29 July 2020. (b) Geographical distribution of cumulative number of deaths of the SARS-CoV-2 disease in West Africa as 29 July 2020.

**Fig. 3.**
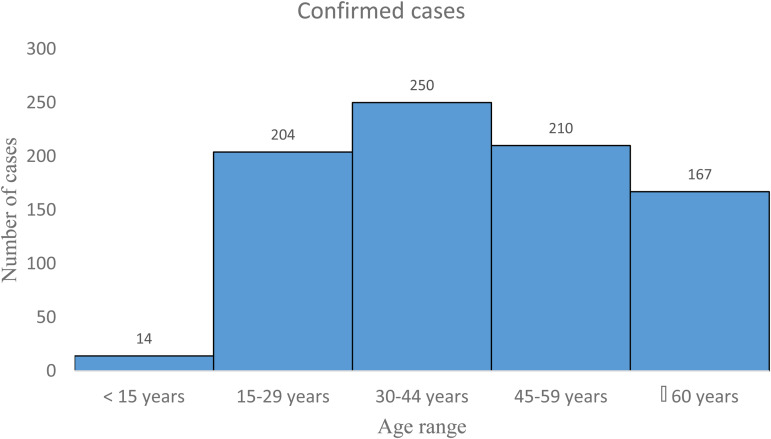
Distribution by age group of COVID-19 cases in Burkina Faso from 9 March to 21 June 2020 (*N* = 845). *Source*: Ministère de la santé Burkina Faso, Sitrep 115. https://www.humanitarianresponse.info/sites/www.humanitarianresponse.info/files/documents/files/sitrep_covid-19_ndeg_115.pdf.

We conducted reviews for a broad range of public health topics related to SARS-CoV-2 focusing on the epidemiology, clinical manifestations and mode of transmission, factors that may influence the circulation of the virus. Initially, we conducted a systematic literature search of electronic databases (PubMed, ScienceDirect, Google Scholar and EMBASE) and pre-print servers (medRxiv and bioRxiv). In addition to the articles, we also searched data from WHO, some international organisations and SitReps on COVID-19. Then we used all this information to describe the situation in West Africa based on demographics, lifestyles, climate, chronic diseases and availability of resources. The research was conducted from March to July 2020. A total of 531 articles were reviewed and all articles that were deemed irrelevant to our review were excluded. These exclusion criteria mainly concerned the geographic location of the study, the epidemiological aspects addressed and the language of publication. The selection was made in three stages: a first stage of selection based on titles, then based on summaries and finally one based on full text. Thus, 40 articles were selected for this review. We have combined the search terms ‘SARS CoV-2 and climatic factors’, OR ‘SARS CoV-2 and chronic diseases’, OR ‘SARS CoV-2 and West Africa’ OR ‘SARS CoV-2 and Africa’. We used data from the COVID-19 Dashboard, a dataset hosted by the Center for Systems Science and Engineering at Johns Hopkins University to plot Figures 1a, b, 2a and b [18]. Figure 3 and Table 1 were plotted using SitRep 115 data on COVID in Burkina Faso. Figure 1a and b were generated by datawrapper, and Figures 2a, b and 3 were generated by Excel 2013.

**Table 1.** Mortality rate of SARS CoV-2 by age group in Burkina Faso as of 21 June 2020 (*n* = 907)

Age range	Number of deaths (% relative to the total number)	Case-fatality rate (%)
<15	0	0
15-29	3 (5.7)	0.3
30-44	4 (7.5)	0.4
44-59	13 (24.5)	1.4
⩾60	33 (62.3)	3.6
Total	53 (100)	5.7

*Source*: Ministère de la santé Burkina Faso, Sitrep 115. https://www.humanitarianresponse.info/sites/www.humanitarianresponse.info/files/documents/files/sitrep_covid-19_ndeg_115.pdf.

### Clinical manifestations and mode of disease transmission

Transmission of coronavirus disease occurs mainly through the inhalation of infectious droplets and aerosols [19] and the incubation period is approximately 3–14 days. The clinical picture of SARS-CoV-2 infection is wide. It can range from an asymptomatic infection to a mild upper respiratory infection or even severe viral pneumonia that can lead to death [20, 21]. Seniors are most likely to develop severe forms [22]. Other non-specific symptoms have been reported, such as fever, cough, myalgia, dermatological damage, dyspnoea with or without diarrhoea and neurological manifestations such as hypogeusia and hyposmia were described [21, 23]. A hyperinflammatory phenomenon called ‘cytokine storm’ is thought to be involved in the occurrence of severe forms [24]. Cytokines are small proteins naturally produced by immune cells to promote ant-inflammatory reaction in order to protect the body from infection [25]. During SARS-CoV-2 infection, there is an excess production of cytokine causing a hyperinflammatory reaction that can lead to death by cytokine shock [24]. All patients should be quarantined and mechanical ventilation in the intensive care unit is mandatory for the management of severe cases. Secondary bacterial infections can also occur, which can lead to pneumonia [26]. There is little data on the clinical manifestations of COVID-19 in West Africa. However, according to a study of 32 COVID-19 patients in Nigeria, no patient presented to hospital under critical conditions. In total, 75% were in moderately severe condition while 16% were asymptomatic. The most common symptoms were fever (59%) and dry cough (44%). Anosmia (loss of smell) and ageusia (loss of taste) were present in 19% of patients [27].

### Epidemiological situation of SARS-CoV-2 in West Africa

The epidemiological situation of SARS-CoV-2 disease in West Africa is marked by a continuous increase in the numbers of confirmed cases with rather disparate situations (Fig. 1a and b) [28]. After a first case notified on 27 February 2020 in Nigeria, West Africa, had on 29 July 2020, 131 049 cases confirmed by polymerase chain reaction (PCR), 88 305 recoveries and 2102 deaths [12]. Nigeria is the most affected country in terms of registered death with 41 804 confirmed cases and 868 deaths followed by Ghana (34 406 confirmed cases and 168 deaths) and Cote d'Ivoire (15 713 confirmed cases and 98 deaths). Among the least affected countries we can cite Gambia (326 confirmed cases and 8 deaths), Togo (896 confirmed cases and 18 deaths) and Chad (926 confirmed cases and 75 deaths) (Fig. 2a and b) [12]. If we analyse in more detail the situation in Burkina Faso, the first two cases were highlighted on 9 March 2020: a couple returning from France, precisely in the city of Mulhouse, where they participated in an evangelist event gathering several tens of thousands of people from 17 to 24 February 2020 and which constitutes the main cluster of COVID-19 in France. In this country young people under 15 are the least affected with only 14 confirmed cases (Fig. 3). Table 1 shows the death rate of SARS-CoV-2 by age group in Burkina Faso as of 21 June 2020. If we take the case of Nigeria, the most affected country in West Africa, a study described that 94% of patients had a recent travel history or contact with a confirmed case and 53% of patients were over 40 years old [27]. Another study reported a case fatality rate from COVID-19 of 2.8%. Lagos State was the most affected with more than 50% of positive cases. In a survey conducted among the population, only 29.0% would agree to be vaccinated if a vaccine against COVID-19 was available [29, 30].

In West Africa the number of confirmed cases is probably largely underestimated, given the limited means of diagnosis in some countries [31]. The diagnosis is based on reverse-transcription (RT)-PCR using a nasopharyngeal swab. Two genes are targeted for the detection of SARS-CoV-2 RNA: the ORF1 gene and the N gene [32]. However, there's approximately a false negative rate of 20% [33].

The low population density in West African countries could probably limit the spread of COVID-19. Indeed, with the exception of Nigeria (220 people/km^2^), the population density of West African countries is relatively low (Burkina Faso: 72 people/km^2^, Niger: 16 people/km^2^, Mali: 15 people/km^2^) compared to European countries (France: 105 people/km^2^, Italy: 207 people/km^2^, United Kingdom: 267 people/km^2^) and Asians countries (China: 144 people/km^2^, Republic of Korea: 516 people/km^2^, Hong Kong: 6510 people/km^2^) [34–36]. Nevertheless, the presence of large urban areas and highly populated capital is a factor favouring the spread of the disease. This is, for example, the case of Lagos in Nigeria with around 6871 people/km^2^ [37].

#### Preventive measures against SARS-CoV-2

The WHO encourages countries to carry out widespread testing, to apply social distancing and to respect barrier measures [38]. Also, the experiences of China and South Korea teach us that rapid and systematic detection of suspected cases, the wearing of masks, as well as studied containment and compliance with barrier rules (hand washing and social distancing) could help to significantly reduce the spread of the disease [39, 40].

In West Africa majority of governments have quickly taken a number of measures to limit the spread of the disease. Among these measures we can cite: (i) border closure; (ii) quarantine and self-containment of contacts of cases; (iii) dissemination of prevention messages calling on the population to wear a mask, to comply with barrier measures and social distancing; (iv) introduction of a curfew in certain countries (Burkina Faso, Cote d'Ivoire, Mali, Senegal, Niger and Guinea) and (v) closure of markets and places of worship.

However, the population struggles to comply with certain measures such as the closing of shops and the travel limitations. In fact, 92.4% of people active in West Africa work in the informal sector as trading and businesses, transport and restoration. These jobs are not subject to any national legislation and are not subject to any social protection [41]. All these people are forced to go to work every day in order to support their families and in the absence of robust economic packages to help them to stay at home physical distancing measures are doomed to failure. Otherwise million people in West Africa lack access to clean water and communal water and sanitation facilities are not always accessible which makes certain hygiene measures recommended to fight against the SARS-CoV-2 epidemic difficult. For example, 63% of people in urban areas, or 258 million people, lack access to handwashing [42]. According to UNICEF data from 2017, the majority of people did not have basic handwashing facilities available at home. Only Ghana, Mali, Mauritania and Nigeria were above the global average of 60% of people with access to basic facilities. The situation was particularly bad in small countries such as Benin, Gambia, Guinea-Bissau, Liberia and Togo where at least three-quarters of the population had no handwashing facility at home [43]. Quality masks are not always accessible to the population. Some countries (Senegal, Nigeria and Mali) have invested in the production and local distribution of masks [44]. However, the quality of these masks is often lacking. For example, in Benin buying a quality mask in pharmacies subsidised at 30 cents is still too expensive for some people. People then flock to masks sold on the street at half price, with a single layer of fabric and without any filter [45]. The average size of households in certain West Africa countries is estimated at 7.2 members per household which mean that several family generations live and interact under the same roof (concession) [46]. This makes it difficult to comply with self-containment and distancing measures. The difficulty in applying all these preventive measures could be a factor favouring the spread of the virus in these countries.

### Case management

The health situation in West African countries is difficult, in particular due to the extremely poor access of populations to health services. This weakness was exposed by the 2014/2015 Ebola virus epidemic in West Africa. This epidemic has revealed significant gaps in the capacity and preparedness to respond effectively to critical health events in affected countries [47]. In a country like Burkina Faso we have six university hospitals for around 20 244 079 inhabitants and one doctor for 12 000 inhabitants [48]. The management of patients with severe forms of COVID-19 requires resuscitation beds and respirators. Most African countries do not have enough of these essential care materials. It is difficult to obtain exact numbers for all countries but some are nevertheless available. For instance, there are only 15 resuscitation beds in Burkina Faso, 40 in Mali and 80 in Senegal. There are only 4 respirators in Togo, 5 in Niger, 11 in Burkina Faso, 20 in Côte d'Ivoire, 56 in Mali, 80 in Senegal and around 400 in Nigeria [49]. The hospital's capacity could quickly be exceeded. So, it is therefore necessary to anticipate the equipment of hospitals for the management of cases. Certain non-specific symptoms of SARS-CoV-2 such as fever and headache resemble that of malaria, hence the need for a differential diagnosis with malaria so as not to miss certain cases of SARS-CoV-2.

#### Problematic of the management of asymptomatic cases

In the strategy implemented to limit the spread of SARS-CoV-2, there is the quarantine of contacts of cases. All people who have been in contact with a confirmed case are quarantined and followed up for 2 weeks. During these 2 weeks, everyone who develops symptoms is tested and managed; and anyone who does not show symptoms after 2 weeks is released from quarantine without being tested as is the case of Burkina Faso. Among these asymptomatic people out of quarantine, there could be asymptomatic carriers of the disease. The literature reports that people with asymptomatic forms of the disease can infect others and this transmission is estimated to be approximately 10% of transmission events [50]. This capacity for transmission of SARS-CoV-2 by asymptomatic carriers could thus increase the risks of spread of the disease in the population. It should therefore be wise to test all contacts of cases without exception after quarantine but this need is faced with the limitation of diagnostic tests available. For example at the beginning of the epidemic, there was only one laboratory (National Influenza Reference Laboratory) for confirmation of the cases for all the Burkina Faso, located in Bobo-Dioulasso, the second city in the country, located 365 km from the capital. Today, four other laboratories have been equipped in Ouagadougou and Bobo-Dioulasso to carry out diagnostic tests, thereby improving diagnostic capacities.

### SARS-CoV-2 and co-morbidities

Several studies in China and Italy tend to confirm that co-morbidities increase the risk of developing serious or even fatal forms of SARS-CoV-2 infection [51–53]. A study on 1099 patients with COVID-19 in China, reported that 173 patients suffering from severe forms had the following comorbidities: high blood pressure (23.7%), diabetes mellitus (16.2%), coronary heart disease (5.8%) and cerebrovascular disease (2.3%) [52].

In West Africa, the most common chronic diseases encountered in the population are diabetes mellitus and high blood pressure [54].

#### Diabetes mellitus

Diabetes mellitus is a public health problem in sub-Saharan Africa. According to the WHO estimates, there will be a significant increase in the prevalence of diabetes in developing countries by 2025, which is expected to house 75% of the world's diabetic patients. In sub-Saharan Africa, the number of adults over the age of 20 with a body mass index greater than 25 kg/m^2^ increased from 28 million in 1980 to 127 million in 2015 [55, 56]. During this same period, the prevalence of diabetes mellitus increased considerably in Africa (3.1% in 1980 to 7.1% in 2014) which is higher than that in many European countries [55]. Although there are few data on the prevalence of diabetes in West Africa, these data indicate that it is an important problem to take into account regarding the SARS-CoV-2 pandemic, the rate being 3% in Benin; in 6% Mauritania, 5% in Ivory Coast and 4.2% in Burkina Faso [57–59].

#### High blood pressure

High blood pressure is defined as a systolic pressure of 140 mmHg or more or a diastolic pressure of 90 mmHg or more [60]. The prevalence of high blood pressure is increasing in sub-Saharan Africa, and has become similar to that seen in high-income African countries [61, 62]. This prevalence varies between 16% and 40% among adults aged 18 and over; and exceeds 60% in people aged 65 and over in some studies [63, 64]. There are high proportions of people with high blood pressure who are unaware of their status (>70%), poor medication compliance (>80%) or of hypertensive patients who are not controlled under treatment (30–80%) [63].

The two co-morbidities mentioned above could lead to an increase in the number of severe cases of SARS-CoV-2 in West African during this pandemic. Also, many people in sub-Saharan Africa live with chronic pathologies without knowing it either through ignorance of the symptomatology or through lack of regular health check-ups. It would therefore be appropriate to strengthen the monitoring of people living with chronic pathologies during this period of SARS-CoV-2 pandemic.

#### SARS-CoV-2 and people living with HIV

There has not yet been any scientific evidence that people living with HIV are at higher risk of contracting COVID-19 or developing severe forms of the disease. However, HIV-infected people are at higher risk of cardiovascular and/or metabolic disorders, as a consequence of chronic immune activation and ARV drugs exposure. They are more likely to contract COVID-19 and develop severe forms. With the exception of Niger (0.3%), Burkina Faso has one of the lowest rates of people living with HIV in the West African sub region (0.7%). Ivory Coast has the highest rate with 2.6% followed by Togo with 2.3%, Ghana with 1.7%, Mali with 1.4% and Benin with 1% [65].

### SARS-CoV-2 and climatic factors

SARS-CoV appeared in China in Foshan, Guangdong province, in November 2002, while SARS-CoV-2 appeared in Wuhan, Hubei province in December 2019 [66, 67]. In China, November and December are the coldest months of the year [68]. Low rainfall was also noted in these two regions during both of these periods of SARS outbreaks. Low temperatures generally constitute conditions favouring the circulation of viruses [68]. Cold accompanied by dry winds would be even more favourable to the survival of viruses compared to cold alone. Indeed, under these conditions, viral particles dried in the air, survive for a long time in the circulating air [69–71]. In addition, cold causes a decrease in immunity in humans, notably by reducing blood supply and therefore a decrease in the supply of immune cells to the nasal mucosa [72]. Low humidity would reduce the ability of cell lashes in the airways to remove viral particles [72]. It can also cause drying of the nasal mucus, thus weakening the nasal cavity; which will make the entire upper respiratory tract vulnerable to viral infection [68]. In a study of 100 Chinese cities, Wang *et al*. found that high temperature and high humidity significantly reduced transmission of SARS CoV-2 [73].

With the exception of the Mediterranean and South Africa, most countries on the African continent have a tropical climate. Temperatures, generally high, vary little throughout the year [74]. In the Sahelian zone the hottest season is between April and June and for the Guinean zone, it is between January and March [75]. During hot periods temperatures can reach 45 °C in some areas; this is the case in Burkina Faso, Niger and Mali [76–78]. These high heats could be a factor contributing to slow the spread of the SARS-CoV-2 in these countries.

The West African rainfall regime is linked to the seasonal movement of the intertropical convergence zone. The semi-arid zone, which essentially comprises the Sahelian and Sahelo-Saharan strip, is marked by a single rainy season between July and September. Further south, the alternation of two rainy seasons and two dry seasons marks the climate of the countries of the Gulf of Guinea [74]. These periods are also marked by an increase in humidity. The arrival of the rainy season in certain West Africa countries, moreover, will increase the humidity, which combined with heat could further reduce the spread of SARS CoV-2.

### SARS-CoV-2 and demography

**Fig. 4.**
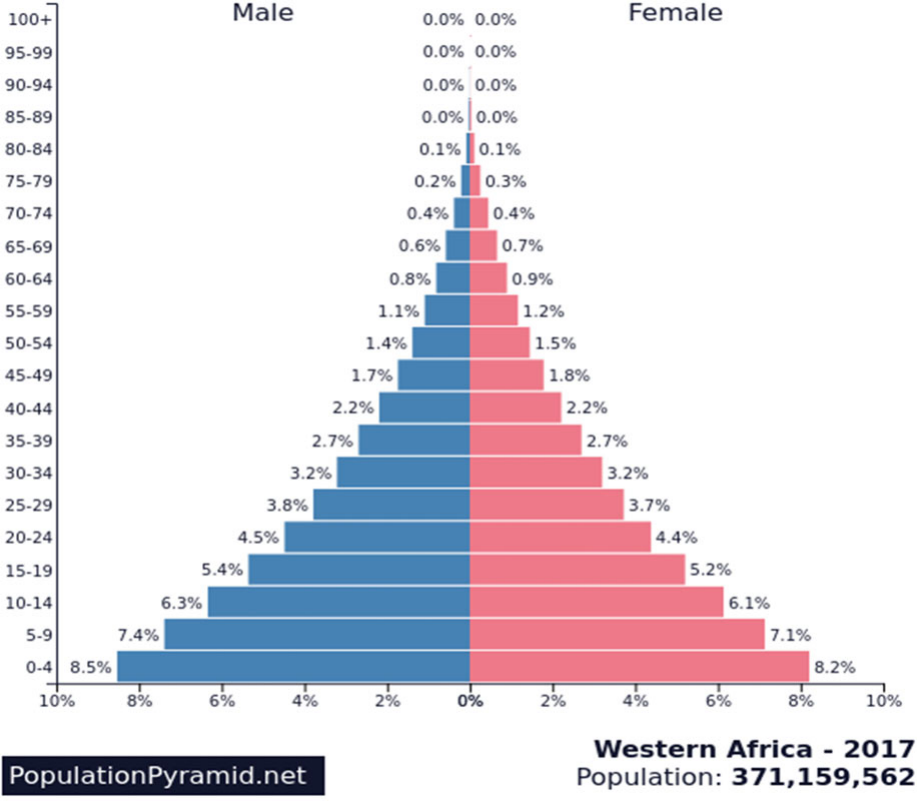
Pyramid of age, West Africa's population. *Source*: PopulationPyramid.net. https://www.populationpyramid.net/western-africa/2017/.

West Africa's population is predominantly young. Out of a population estimated at 371 159 562 in 2017, over 64% are under the age of 24 (Fig. 4) [79, 80]. The youthfulness of the West Africa's population could be a factor limiting the occurrence of severe forms of SARS-CoV-2 infection. Indeed, the epidemiological situation of COVID-19 infection in China and Italy supports the hypothesis that the age factor plays an important role in the occurrence of severe forms of the disease [22]. The fatality rate higher in Italy than in China could be explained in particular by the fact that the Italian population is one of the oldest in the world with approximately 22% of the population aged 65 and over [81]. This could contribute, in part, to the high case fatality rate compared to other countries. In the African context, intra-family transmission of SARS-CoV-2 may be linked to the fact that several families and several generations live and interact under the same compound. In addition, the youngest live with the oldest within the extended family unit, making the risk of contamination of the elderly by the young higher.

## Conclusion

Although the spread of the SARS-CoV-2 epidemic in West Africa is relatively slow compared to European, North American or Asian countries, vigilance must remain. West Africa has already registered more than 131 049 cases confirmed by PCR with 2102 deaths. This figure, however, falls far short of those recorded by European countries such as France, Spain, UK and Italy. The West African's population way of life and the large number of people working in the informal sector make it difficult to apply preventive measures, namely physical distancing and confinement. In order to be able to effectively combat the spread of the virus, it is essential to develop a strategy based on the protection of people at risk, namely the elderly and people living with chronic diseases. Mass screening targeting mainly people at risk and their entourage would allow early detection of cases and quarantine to breaking the chain of transmission and protecting the more vulnerable ones. A local production of protective masks could also be an alternative to the shortage of masks that the world is currently facing. Experiences from past epidemics (such as Ebola, dengue, yellow fever and Lassa fever) have increased laboratory and surveillance capabilities across the African continent which allows West Africa to be better prepared to face the COVID-19 pandemic. For example, in Burkina Faso, the epidemic of Ebola virus disease has led to the creation of a national reference laboratory for viral haemorrhagic fevers and the training of several health workers in the management of epidemics. It is to be hoped that this epidemic will boost care and detection capacities in the near future.

## Data Availability

The data that support the findings of this study are openly available in *The Lancet* at https://www.thelancet.com/journals/laninf/article/PIIS1473-3099(20)30120-1/fulltext [18]. We also used data from the ministry of health of Burkina Faso available in free access at https://www.humanitarianresponse.info/sites/www.humanitarianresponse.info/files/documents/files/sitrep_covid-19_ndeg_115.pdf.
